# Generative AI in physical education and health: a narrative review and conceptual framework for interdisciplinary thematic learning

**DOI:** 10.3389/fpubh.2026.1842782

**Published:** 2026-06-03

**Authors:** Gewenjin Zhu, Xi Dai, Suqi Jiang, Zili Wang, Mengyuan Liang, Yuchen Pan, Yuliu Tao

**Affiliations:** 1 School of Physical Education, Soochow University, Suzhou, China; 2 Dongwu Think Tank, Soochow University, Suzhou, China

**Keywords:** conceptual framework, generative artificial intelligence, interdisciplinary thematic learning, narrative review, physical education and health

## Abstract

Generative artificial intelligence (GenAI) has created new possibilities for teaching and learning, yet its pedagogical role in physical education and health (PEH) remains underdeveloped. Current AI-related research in PEH has largely focused on performance-oriented and technical applications, such as motion analysis, skill evaluation, and training monitoring, with less attention to curriculum design, interdisciplinary learning, and teacher-mediated pedagogical use. This narrative review synthesizes literature on GenAI in PEH and related interdisciplinary or thematic learning contexts to examine how GenAI may support interdisciplinary thematic learning in PEH. The review highlights an emerging shift from AI as a technical or analytical tool toward GenAI as a pedagogical resource for lesson planning, assessment design, feedback, inquiry support, and knowledge integration. Based on this synthesis, the study proposes a pedagogical framework that positions GenAI as a teacher-mediated resource for connecting bodily practice, health knowledge, reflective inquiry, and social participation. The framework includes four core dimensions: curriculum and goal alignment, content organization and task design, inquiry and learning process support, and assessment and adaptive adjustment. It also emphasizes human-AI collaboration, teacher judgment, embodied observation, and ethical use as necessary conditions for meaningful implementation. The review suggests that GenAI may support PEH when it is integrated carefully into pedagogically designed, context-sensitive, and health-promoting learning processes. Future empirical research, particularly design-based studies and classroom interventions, is needed to test and refine the framework in authentic PEH settings.

## 1 Introduction

In recent years, the emergence of large-scale generative models, such as ChatGPT and Sora, has marked a significant shift in the development of artificial intelligence. Generative artificial intelligence (GenAI) is increasingly reshaping knowledge production and learning across domains, with education identified as one of the most affected fields (1). GenAI has been applied to instructional support, personalized learning, feedback generation, and assessment design, while also raising concerns regarding pedagogical integration, reliability, and ethical use (2). These developments suggest that the educational value of GenAI depends not only on content generation, but also on its alignment with pedagogical processes. This issue becomes particularly salient in learning contexts that require the integration of multiple knowledge sources and perspectives. In such contexts, GenAI can support interdisciplinary learning by facilitating knowledge translation across domains and promoting shared understanding (3). Rather than functioning solely as a content-generation tool, GenAI may act as a mediating mechanism that supports knowledge integration and inquiry processes.

This perspective is especially relevant in physical education and health (PEH). Contemporary PEH extends beyond movement performance and is increasingly understood as a multidimensional learning domain integrating physical activity, health knowledge, and personal and social development. Meaningful learning in PEH requires students to connect bodily practice with cognitive understanding, reflection, and social engagement (4). This perspective is also supported by evidence linking physical activity with cognitive and health-related outcomes in children and adolescents (5). Interdisciplinary thematic learning is therefore closely aligned with the integrative nature of PEH. However, current research on artificial intelligence in PEH remains largely focused on performance-oriented and technical applications, such as motion analysis, skill evaluation, and training monitoring (6). While these studies contribute to improving performance and health outcomes, they tend to conceptualize AI as an analytical tool, with comparatively limited attention to its role in pedagogy, curriculum design, and interdisciplinary learning.

This imbalance points to a gap in understanding how GenAI can be meaningfully integrated into PEH at the instructional and curriculum levels. In particular, there is a lack of integrative frameworks that connect GenAI capabilities with pedagogical processes and interdisciplinary learning goals in PEH contexts. To address this gap, the present study adopts a narrative review approach to synthesize research on GenAI in PEH and related interdisciplinary learning contexts. Based on this synthesis, the study develops a pedagogical framework that links GenAI-supported learning inputs, core pedagogical processes, and intended learning outcomes in PEH. The aim is to clarify how GenAI may be pedagogically integrated into interdisciplinary thematic learning in PEH while preserving teacher judgment, embodied participation, and health-promoting educational purposes.

## 2 Method

### 2.1 Review design and aims

This study adopted a narrative review design to examine the emerging role of GenAI in PEH, with particular attention to its pedagogical relevance for interdisciplinary thematic learning. A narrative approach was selected because the topic spans two related bodies of literature: GenAI-supported PEH and physical education contexts, and GenAI-supported interdisciplinary or thematic learning. These areas involve diverse forms of evidence, including empirical studies, review articles, and conceptual analyses. This approach enables the integration of heterogeneous literature and supports the development of a conceptually informed pedagogical framework. The aim of the review was to identify key themes, gaps, and pedagogical implications in the literature and to inform the development of a pedagogical framework for GenAI-supported interdisciplinary thematic learning in PEH.

### 2.2 Search strategy

A structured literature search was conducted across PubMed, Web of Science, Scopus, and Embase. The search did not impose a fixed start date to ensure comprehensive coverage and was updated to include publications up to May 5, 2026. Search terms were defined *a priori* and combined two concept groups: GenAI and PEH-related or interdisciplinary thematic learning contexts. The detailed search strategy for each database is presented in Table 1. In addition to database searches, relevant studies were identified through backward and forward citation tracking and manual checking of key references.

**Table 1:** Database search strategy.

Database	Search string
PubMed	(“generative artificial intelligence”[Title/Abstract] OR “generative AI”[Title/Abstract] OR GenAI[Title/Abstract] OR ChatGPT[Title/Abstract]) AND (“physical education”[Title/Abstract] OR “physical education and health”[Title/Abstract] OR PEH[Title/Abstract] OR “interdisciplinary learning”[Title/Abstract] OR “thematic learning”[Title/Abstract] OR “interdisciplinary teaching”[Title/Abstract] OR “interdisciplinary education”[Title/Abstract] OR “integrated curriculum”[Title/Abstract] OR “curriculum integration”[Title/Abstract])
Web of Science	TS = (“generative artificial intelligence” OR “generative AI” OR GenAI OR ChatGPT) AND TS = (“physical education” OR “physical education and health” OR PEH OR “interdisciplinary learning” OR “thematic learning” OR “interdisciplinary teaching” OR “interdisciplinary education” OR “integrated curriculum” OR “curriculum integration”)
Scopus	TITLE-ABS-KEY (“generative artificial intelligence” OR “generative AI” OR GenAI OR ChatGPT) AND TITLE-ABS-KEY (“physical education” OR “physical education and health” OR PEH OR “interdisciplinary learning” OR “thematic learning” OR “interdisciplinary teaching” OR “interdisciplinary education” OR “integrated curriculum” OR “curriculum integration”)
Embase	(‘generative artificial intelligence’:ti,ab OR ‘generative ai’:ti,ab OR genai:ti,ab OR chatgpt:ti,ab) AND (‘physical education’:ti,ab OR ‘physical education and health’:ti,ab OR peh:ti,ab OR ‘interdisciplinary learning’:ti,ab OR ‘thematic learning’:ti,ab OR ‘interdisciplinary teaching’:ti,ab OR ‘interdisciplinary education’:ti,ab OR ‘integrated curriculum’:ti,ab OR ‘curriculum integration’:ti,ab)

### 2.3 Eligibility criteria and study selection

Two reviewers (GZ and XD) independently screened the titles and abstracts of the identified records and excluded clearly irrelevant articles. For records that appeared potentially eligible, full texts were retrieved and assessed against the eligibility criteria. Disagreements or uncertain cases were discussed with a third author (SJ) until consensus was reached.

Studies were included if they met one of the following criteria: (1) examined the application of GenAI or related AI technologies in PEH, physical education, physical activity, or school-based movement and health learning contexts; or (2) examined the application of GenAI or related AI technologies in interdisciplinary learning, thematic learning, curriculum integration, or human-AI interaction in educational settings. Eligible studies could include empirical studies, review articles, and conceptual papers published in English. Studies were excluded if they focused on non-educational applications of AI, were purely technical without pedagogical relevance, or were unrelated to PEH, physical education, physical activity learning, or interdisciplinary/thematic learning. Studies focusing mainly on patient education materials, disease-specific health education, clinical communication, or health professional training, such as medicine, nursing, or dentistry, were also excluded. Non-peer-reviewed publications, including dissertations, theses, conference papers, book chapters, and preprints, were excluded.

### 2.4 Data synthesis and framework development

A narrative and thematic synthesis approach was used. Relevant studies were reviewed to identify recurring concepts related to pedagogical processes, curriculum design, interdisciplinary learning, PEH-specific learning contexts, and human-AI interaction. These concepts were first extracted and coded, and then grouped into broader thematic categories. The synthesis distinguished between studies directly focusing on PEH contexts and those addressing broader interdisciplinary or thematic learning perspectives. Core insights from PEH-related studies were used to ground the framework in domain-specific teaching contexts, while complementary evidence from interdisciplinary and pedagogical studies informed the theoretical structure of the framework. The framework was developed by integrating three elements: learning inputs, core pedagogical processes, and intended learning outcomes. Learning inputs included student characteristics, teacher and curriculum inputs, and interdisciplinary resources. Core pedagogical processes included curriculum and goal alignment, content organization and task design, inquiry and learning process support, and assessment and adaptive adjustment. Intended learning outcomes included movement competence and embodied learning, health literacy, reflection and decision-making, and social participation, collaboration and values. Human-AI collaboration was treated as a cross-cutting principle across the framework, emphasizing teacher mediation, student participation, and GenAI-assisted support within pedagogically guided learning processes. Given the narrative and integrative nature of this review, a formal critical appraisal of the included studies was not conducted. This decision was consistent with the purpose of the review, which was to develop a conceptually informed pedagogical framework, rather than to evaluate intervention effectiveness, compare study quality, or synthesize empirical effect sizes. The findings should therefore be interpreted as a theoretically informed synthesis that may be subject to potential selection bias.

## 3 Results

### 3.1 Overview of the included literature

**Figure 1:**
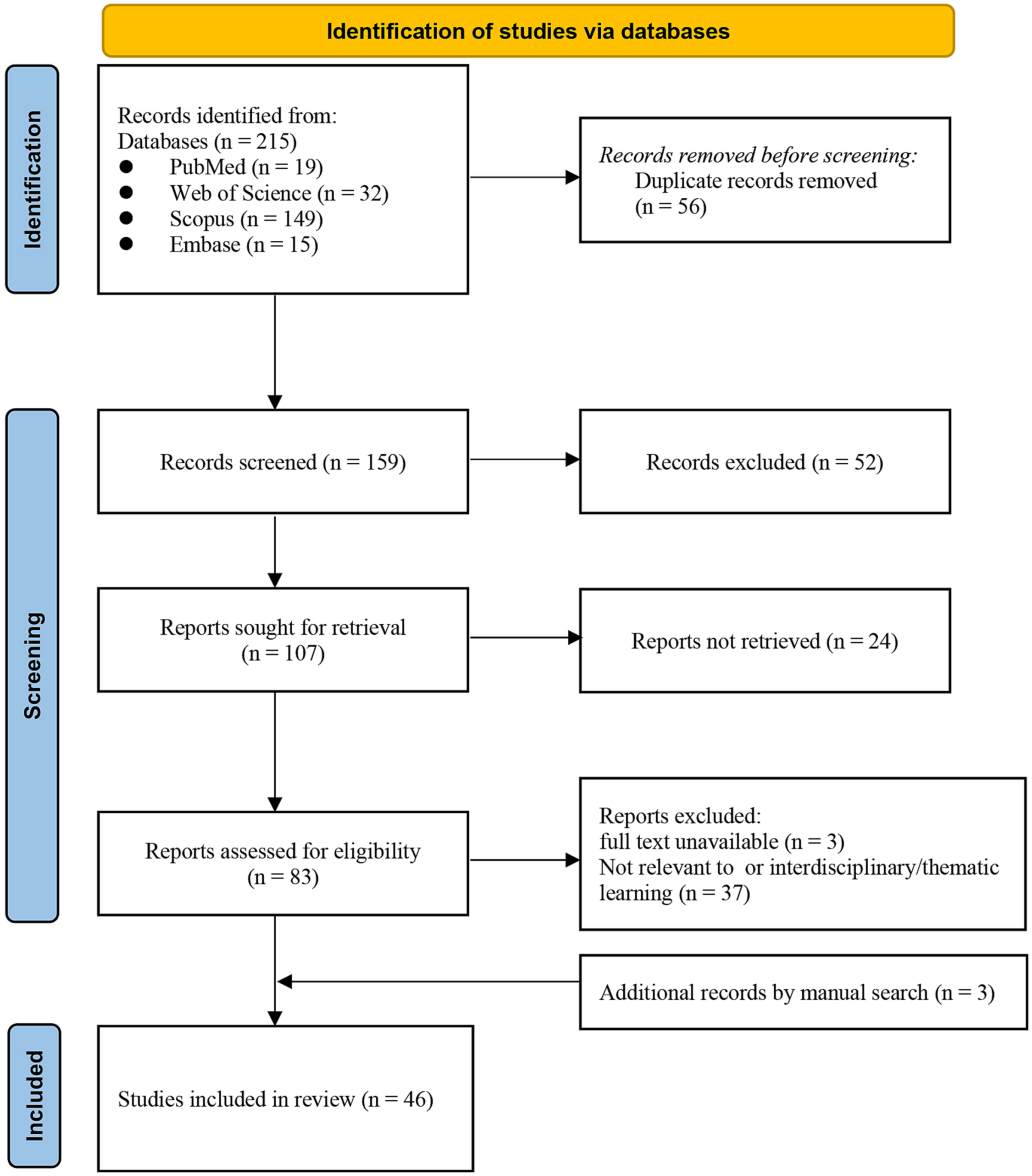
Flowchart of study selection.

The study selection process is presented in Figure 1. A total of 46 studies were included in this narrative review. Among them, 23 studies focused on PEH, physical activity learning, sport-related instruction, kinesiology, or PE teacher education, while 23 studies addressed GenAI-supported interdisciplinary or thematic learning in broader educational contexts. The included literature comprised empirical, intervention, design-based, qualitative, review, conceptual, theoretical, framework-based, and practice-oriented studies. For narrative synthesis, the included literature was organized into seven thematic areas, as summarized in Table 2.

**Table 2:** Thematic synthesis of included literature.

Thematic area	Educational contexts	Main role of GenAI/AI	Relevance to the framework
Teacher planning, instructional design, and assessment in PEH (7–12, 25)	K-12 PE, HPE, PE teacher practice	Lesson planning, assessment generation, activity adaptation, prompting support	Supports curriculum alignment, task design, and teacher-mediated use of GenAI
PEH teacher competence and AI-related pedagogical knowledge (13–17, 40, 67)	PE teacher education, university PE teachers, HPE teacher education	AI literacy, Intelligent-TPACK, ethical awareness, professional learning	Highlights the need for teacher agency, professional judgment, and ethical human-AI collaboration
GenAI-supported PEH learning, feedback, and tactical/skill development (18–23, 68)	Basketball, badminton, handball, football, college fitness classes	Rule explanation, corrective feedback, tactical interpretation, scenario-based learning	Supports inquiry, feedback, adaptive adjustment, and learning process support
Inclusive and health-promoting physical activity (24, 69)	Home-based physical activity, neurodivergent children, sports and health education	Simplified instructions, UDL-informed adaptation, health decision-making support	Links GenAI with health literacy, equitable participation, and public health-oriented PEH
Interdisciplinary curriculum development and knowledge integration (3, 26–32, 70–72)	Teacher education, doctoral education, undergraduate interdisciplinary learning	Brainstorming, boundary crossing, knowledge integration, self-regulated scaffolding	Supports GenAI as a mediating mechanism for interdisciplinary learning
Environmental, sustainability, and systems-based thematic learning (33–37)	Environmental education, geography, STEM, sustainability	Environmental simulation, systems analysis, geographic storytelling, personalized support	Shows how GenAI can support complex real-world thematic learning relevant to health and public well-being
AI literacy, ethics, creativity, and human-centered use (38, 39, 41, 42, 73–75)	School education, higher education, social work, art/design, social studies, PE	AI literacy, ethical reflection, creative co-design, critical evaluation	Emphasizes responsible, reflective, and human-centered GenAI integration

AI, artificial intelligence; GenAI, generative artificial intelligence; PEH, physical education and health; PE, physical education; HPE, health and physical education; TPACK, technological pedagogical content knowledge; UDL, universal design for learning; STEM, science, technology, engineering, and mathematics.

The PEH-related studies show a gradual shift from early conceptual discussions of ChatGPT and AI-powered chatbots toward more concrete pedagogical applications and empirical investigations. Early studies mainly discussed the implications of ChatGPT for health and physical education teacher education, lesson planning, assessment, academic integrity, digital equity, and professional judgment. More recent studies examined GenAI-supported content knowledge, tactical instruction, skill feedback, physical fitness learning, inclusive physical activity, and PE teacher professional development.

The interdisciplinary and thematic learning studies covered a broader range of contexts, including teacher education, environmental education, sustainability education, geography, STEM, art and design, social work, middle school education, and social studies. Across these studies, GenAI was commonly positioned as a tool for knowledge integration, curriculum design, problem reframing, boundary crossing, personalized support, and reflective human-AI collaboration. These two evidence groups are complementary: the PEH-related studies clarify the specific pedagogical possibilities and constraints of GenAI in embodied and health-oriented learning contexts, while the interdisciplinary and thematic learning studies explain how GenAI may support cross-disciplinary meaning-making and inquiry.

### 3.2 GenAI as a pedagogical support tool in PEH

Although AI-related research in PEH has largely focused on performance-oriented and technical applications (6), the GenAI-focused PEH studies included in this review suggest an emerging pedagogical turn. These studies extend the role of AI beyond movement analysis, performance monitoring, and exercise tracking by exploring GenAI as a resource for lesson planning, assessment design, feedback, professional learning, and teacher-mediated instructional support (7–24).

Several practice-oriented studies discussed how PE teachers may use ChatGPT or other GenAI tools to generate lesson plans, design assessments, adapt activities, differentiate instruction, and support student motivation (7–12, 25). Studies on GenAI-supported lesson planning and assessment design proposed checklists, prompt models, and structured procedures for creating PE lesson plans, rubrics, checklists, and rating scales (8, 10, 11). They also emphasize that AI-generated outputs should be reviewed, adapted to local contexts, and aligned with curriculum standards and students’ developmental needs (7, 8, 10–12). Another group of studies examined PE teacher competence and AI-related pedagogical knowledge. Empirical work involving PE teacher education students and university PE teachers indicates that GenAI adoption and AI-related competence are associated with self-regulation, Intelligent-TPACK, ethical awareness, and professional competence (16, 17). Conceptual and reflective studies further suggest that PE teachers’ professional roles may need to evolve as GenAI becomes more integrated into teaching, assessment, and professional learning (12–15). These findings indicate that effective GenAI use in PEH requires suitable access to AI tools, together with teachers’ ability to evaluate AI outputs, align them with pedagogical purposes, and use them ethically.

A further group of studies focused on student learning, feedback, and skill development. Some examined theoretical learning in sport, such as basketball content knowledge and badminton rule learning (19, 20). Others examined skill-based or tactical learning, including ChatGPT-supported handball feedback, AI-assisted football tactical instruction, and GenAI-supported scenario-based physical fitness learning (18, 21–23). These studies suggest that GenAI can support PEH learning through rule explanation, corrective feedback, tactical interpretation, and adaptive learning scenarios. However, GenAI appears most useful when embedded within structured pedagogical models, such as reciprocal teaching, scenario-based learning, teacher-supervised feedback, or human-AI collaborative design (18, 21–23). GenAI can therefore be understood as a mediated pedagogical resource that extends teachers’ instructional capacity while continuing to rely on professional judgment, embodied observation, and human guidance.

### 3.3 GenAI-supported interdisciplinary and thematic learning

The interdisciplinary and thematic learning literature provides a broader basis for understanding how GenAI may support knowledge integration across disciplinary boundaries. Interdisciplinary learning requires learners to connect different forms of knowledge, negotiate meaning across disciplinary perspectives, and engage with ill-structured problems. In AI-enabled learning contexts, interdisciplinary competence has also been described as involving knowledge connectivity, critical interdisciplinary analysis, AI-driven innovation, collaborative problem-solving, and adaptive transfer (26). These perspectives suggest that GenAI may support interdisciplinary learning by offering explanations, analogies, alternative viewpoints, and opportunities for iterative human-AI dialogue.

Several included studies directly examined GenAI-supported interdisciplinary learning in higher education. These studies showed that GenAI can help learners access disciplinary knowledge, generate ideas, compare perspectives, and develop interdisciplinary projects or proposals (3, 27–31). Some studies described GenAI as a boundary broker or boundary object that supports interdisciplinary discourse and boundary crossing (29). Others found that GenAI can strengthen disciplinary grounding, although integration across disciplines remains challenging and does not occur automatically (3, 30). This finding suggests that GenAI supports interdisciplinary learning by helping learners clarify concepts, identify connections, and refine ideas across domains.

However, the evidence also indicates that meaningful interdisciplinary learning requires structured pedagogical support. GenAI may improve access to information, but access alone does not ensure deep integration. Studies on GenAI-supported interdisciplinary learning highlight the importance of task design, appropriate prompting, self-regulated scaffolding, collaborative inquiry, and reflection (3, 28, 30, 31). This is consistent with prior review evidence showing that AI can support interdisciplinary learning through interactive, immersive, and personalized experiences, while also requiring careful instructional design and teacher guidance (32). A second group of studies examined GenAI-supported thematic learning in environmental, sustainability, geographical, STEM, and arts-related contexts. These studies showed that GenAI can support environmental literacy, systems thinking, geographic storytelling, scenario-based learning, spatial visualization, creative co-design, and responsible AI literacy (33–39). Such studies are relevant to PEH because they illustrate how GenAI can help learners engage with complex real-world issues, including health, environment, sustainability, public well-being, and everyday decision-making. They also suggest that interdisciplinary thematic learning is most productive when GenAI helps connect scientific, social, ethical, cultural, and practical dimensions of learning.

### 3.4 Pedagogical roles and challenges of GenAI

Across the included literature, GenAI was used in several overlapping pedagogical ways. In PEH and broader educational contexts, it supported lesson planning, assessment design, activity adaptation, curriculum development, and thematic module design. It also functioned as a dialogue partner and cognitive scaffold, helping learners brainstorm ideas, clarify concepts, reframe problems, compare disciplinary perspectives, and refine learning products. In addition, GenAI was used to support feedback and assessment, particularly in PEH skill learning, tactical instruction, formative assessment, and AI-supported course assessment. In interdisciplinary contexts, it further helped learners connect ideas across domains and engage with complex thematic issues.

These pedagogical possibilities were accompanied by several concerns. GenAI outputs may contain hallucinations, factual errors, bias, or contextually inappropriate recommendations, which is especially important in PEH because inaccurate feedback or unsafe activity suggestions may affect students’ physical participation and safety (19, 23, 37, 40). Another concern is that students may rely on AI-generated responses without adequate verification, particularly when they lack disciplinary knowledge or critical evaluation skills (30, 41). In PEH, there is also a specific limitation related to embodiment. GenAI can support explanation, planning, and feedback, but it cannot fully reproduce the tactile, uncertain, and socially situated qualities of real-world physical activity. Because PEH depends on bodily presence, movement experience, and teacher-student interaction, teacher mediation remains essential (15). Ethical and institutional issues, including algorithmic bias, unequal access, academic integrity, privacy, data security, and the need for clear guidance, also remain important (13, 37, 40, 42).

Taken together, the literature suggests that the main gap lies in the lack of pedagogically grounded models explaining how GenAI can be meaningfully integrated into PEH’s multidimensional and interdisciplinary learning processes. PEH-related studies have begun to examine GenAI in lesson planning, assessment, feedback, and teacher development. Interdisciplinary learning studies show that GenAI can support knowledge integration, boundary crossing, and thematic inquiry. These two evidence bases have rarely been connected. The thematic areas summarized in Table 2 therefore informed the development of the framework proposed in the following section. Studies on teacher planning, assessment, and PEH teacher competence informed the teacher-mediated and curriculum-oriented components of the framework. Studies on PEH learning, feedback, inclusive physical activity, and health-promoting participation informed the intended learning outcomes related to movement competence, health literacy, reflection, and social participation. Studies on interdisciplinary curriculum development, thematic learning, AI literacy, ethics, and human-centered use informed the framework’s emphasis on knowledge integration, inquiry support, responsible use, and human-AI collaboration. There remains insufficient guidance on how GenAI can support interdisciplinary thematic learning in PEH while preserving embodied learning, teacher judgment, health-promoting aims, and human-centered pedagogy. This gap provides the rationale for the conceptual framework proposed in the following section.

## 4 A pedagogical framework for GenAI-supported interdisciplinary thematic learning in PEH

Building on the narrative synthesis above, this study proposes a pedagogical framework for GenAI-supported interdisciplinary thematic learning in PEH. The framework conceptualizes GenAI as a pedagogically mediated resource that can support teachers and students in connecting bodily practice, health knowledge, reflective inquiry, and social participation. This position is important because meaningful learning in PEH extends beyond physical performance or health behavior. It involves embodied experience, cognitive understanding, emotional regulation, collaboration, and value formation (43).

The framework responds to two related limitations in current research and practice. On the one hand, AI applications in PEH have often focused on movement analysis, performance monitoring, and technical feedback, with less attention to curriculum design, interdisciplinary content organization, and pedagogical interaction. On the other hand, interdisciplinary teaching in PEH may remain superficial if it simply combines PE with other subjects without a clear mechanism for connecting movement competence, health literacy, scientific understanding, and social learning. Therefore, the framework emphasizes how GenAI can support interdisciplinary thematic learning while preserving the embodied, relational, and health-promoting nature of PEH.

**Figure 2:**
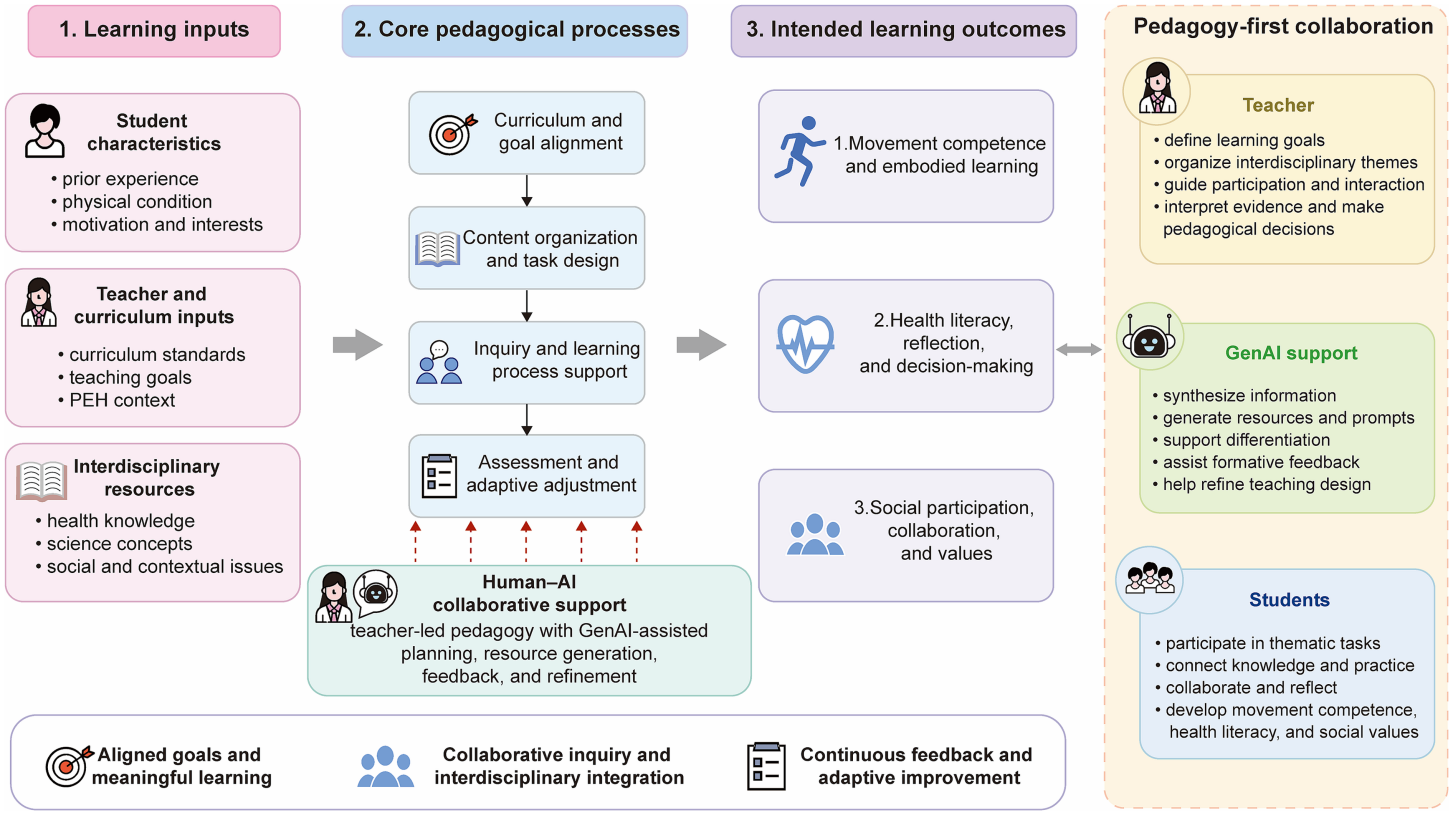
A pedagogical framework for GenAI-supported interdisciplinary thematic learning in physical education and health.

As shown in Figure 2, the framework consists of three main elements: learning inputs, core pedagogical processes, and intended learning outcomes. Learning inputs include student characteristics, teacher and curriculum inputs, and interdisciplinary resources. Core pedagogical processes include curriculum and goal alignment, content organization and task design, inquiry and learning process support, and assessment and adaptive adjustment. These processes are supported by human-AI collaboration, in which teachers, students, and GenAI play distinct but interconnected roles. The framework provides a pedagogical logic for using GenAI in PEH, rather than a technical system model.

### 4.1 Framework rationale and design logic

The framework begins from a pedagogy-first perspective. In PEH, the central question is how GenAI use can serve educational purposes. PEH is characterized by bodily participation, situated interaction, and broader developmental aims. Alongside movement performance, students are expected to develop health literacy, self-regulation, collaboration, and a sense of responsibility in practice (44). These characteristics make PEH especially suitable for interdisciplinary thematic learning, because students need to connect physical activity with scientific concepts, health-related reasoning, emotional experience, and social meaning. From this perspective, GenAI can support the organization of learning. It may help teachers identify relevant themes, generate instructional resources, formulate guiding questions, design differentiated tasks, and organize feedback. It may also help students clarify concepts, compare perspectives, and reflect on their learning. These functions become educationally meaningful when they are guided by curriculum goals, teacher judgment, and students’ embodied participation. This position is consistent with human-centered perspectives on AI in education, which emphasize that the value of AI depends on how it is aligned with educational goals, teacher agency, and meaningful interaction (45). In the proposed framework, GenAI is therefore treated as a mediating resource. It can help connect knowledge, practice, and reflection, but it cannot determine the educational value of learning on its own. The teacher remains central in defining goals, interpreting context, guiding participation, and making decisions about safety, ethics, and values. The four core pedagogical dimensions were derived from this teaching logic. Curriculum and goal alignment clarifies the educational purpose of GenAI-supported thematic learning; content organization and task design translates this purpose into coherent learning activities; inquiry and learning process support guides students’ embodied participation, questioning, and reflection; and assessment and adaptive adjustment helps teachers interpret learning evidence and refine instruction. Together, these four dimensions represent interdependent pedagogical functions that connect GenAI use with the curriculum, learning process, and intended outcomes of PEH.

### 4.2 Core pedagogical dimensions

The first dimension is curriculum and goal alignment. Interdisciplinary thematic learning in PEH should begin with the educational value of the theme and its connection to curriculum purposes. Teachers need to clarify how a theme relates to PEH curriculum goals, students’ developmental needs, and broader health-promoting purposes. For example, themes such as active lifestyles, injury prevention, nutrition and physical activity, mental well-being, environmental health, or digital health literacy can connect movement practice with health knowledge and social understanding. GenAI may support this process by helping teachers organize relevant concepts, identify interdisciplinary links, and translate broad themes into clearer learning objectives. The selection of themes and goals, however, remains a pedagogical decision.

The second dimension is content organization and task design. Interdisciplinary learning depends on how content is structured and how tasks are designed. In PEH, this means linking sports skills, health concepts, scientific principles, and contextual issues in a coherent way. GenAI may assist teachers by generating thematic sequences, scenario descriptions, explanatory materials, and differentiated task options. It can also help teachers design tasks that move from bodily participation to reflection and transfer. For example, a lesson on aerobic exercise may be extended into a thematic inquiry about heart rate, energy expenditure, stress regulation, and lifestyle decision-making. Recent review evidence suggests that GenAI is increasingly used to support instructional design, content generation, and classroom learning activities, indicating its relevance for organizing adaptive and interdisciplinary learning experiences (46).

The third dimension is inquiry and learning process support. In interdisciplinary thematic learning, students should not only receive information, but also ask questions, explore relationships, solve problems, and reflect on experience. This is particularly important in PEH, where learning occurs through movement, perception, communication, and situated engagement. GenAI may support inquiry by generating guiding questions, offering alternative explanations, helping students compare perspectives, and making conceptual links more explicit. It may also support self-regulated learning by helping students plan, monitor, evaluate, and revise their learning processes (47, 48). To preserve students’ active role, teachers need to design inquiry tasks that require students to test ideas through bodily practice, discussion, observation, and reflection.

The fourth dimension is assessment and adaptive adjustment. Assessment in PEH should extend beyond performance indicators. In interdisciplinary thematic learning, assessment should also consider students’ conceptual understanding, health-related reasoning, participation in inquiry, reflective awareness, collaboration, and value development. GenAI may assist teachers by organizing evidence, generating formative feedback, summarizing learning progress, and suggesting areas for refinement. Recent work on assessment redesign in the GenAI era emphasizes the need to align learning outcomes, assessment strategies, and assessment methods, rather than simply rewarding tool-assisted completion (49). In PEH, this means that AI-supported assessment should remain formative, multidimensional, and teacher-mediated. Decisions about how to interpret evidence and adapt instruction remain pedagogical decisions, even when GenAI provides technical support (50, 51).

Together, these four dimensions describe a teaching process in which GenAI supports interdisciplinary thematic learning under pedagogical guidance. The process begins with curriculum and goal alignment, develops through content and task design, unfolds through inquiry and participation, and is refined through assessment and adaptive adjustment.

### 4.3 Human-AI collaboration and teacher mediation

A central feature of the framework is human-AI collaboration. In PEH, teachers, students, and GenAI have different roles. Teachers define learning goals, organize themes, guide participation, interpret evidence, ensure safety, and support students’ emotional and social development. Students participate in movement, inquiry, reflection, and collaboration. GenAI supports planning, resource generation, differentiation, feedback organization, and refinement of teaching design. This division of pedagogical labor is especially important in PEH. GenAI can provide explanations, prompts, and feedback, while teachers interpret students’ embodied responses, classroom dynamics, and learning needs within specific teaching situations. Such situated professional judgment depends on real-time observation, relational understanding, and contextual sensitivity, which cannot be replaced by pre-generated AI outputs. Teacher mediation is therefore central to the use of AI-generated suggestions, which should be treated as provisional resources for professional interpretation.

Human-AI collaboration may still enhance PEH teaching when it is used carefully. Evidence from AI-supported PE environments suggests that AI can support feedback, engagement, and learning motivation when embedded within meaningful teaching activities (52, 53). At the same time, teachers’ readiness and AI-related pedagogical knowledge are critical. Research using AI-related TPACK frameworks indicates that teachers and students may differ substantially in their confidence and preparedness for AI integration (54). This suggests that the effectiveness of GenAI in PEH depends not only on the tool itself, but also on professional development, institutional support, and teachers’ ability to connect AI with pedagogical and content knowledge. For this reason, the proposed framework emphasizes teacher-mediated GenAI use. Teachers should decide when GenAI is useful, how its outputs should be adapted, and whether its suggestions are appropriate for students’ learning conditions and classroom context. In PEH, this mediation has a distinctive embodied and ethical dimension because teaching decisions are continuously shaped by movement observation, participation quality, interpersonal interaction, and health-related responsibility. In this sense, GenAI can extend teachers’ instructional capacity while preserving teacher agency, embodied interaction, and value guidance.

### 4.4 Intended learning outcomes and framework contribution

The framework is oriented toward three broad categories of learning outcomes. The first is movement competence and embodied learning. Students should develop motor skills, movement awareness, and the ability to learn through bodily practice. The second is health literacy, reflection, and decision-making. Students should understand how physical activity relates to health, well-being, risk prevention, and everyday choices. The third is social participation, collaboration, and values. Students should learn to communicate, cooperate, take responsibility, and connect personal health with broader social contexts. These outcomes reflect the integrative nature of PEH. Recent PEH research increasingly frames physical education as a multidimensional educational field concerned with physical well-being, social skills, character development, self-efficacy, motivation, and enjoyment of physical activity (55, 56). The value of GenAI in this framework should be judged by whether it helps teachers and students work toward these broader educational purposes. The framework connects GenAI’s pedagogical affordances with the specific learning logic of PEH. It shows how GenAI may support curriculum alignment, task design, inquiry, feedback, and human–AI collaboration while maintaining attention to embodiment, health promotion, teacher judgment, and student participation. In doing so, the framework provides a conceptual basis for future instructional design and empirical research on GenAI-supported interdisciplinary thematic learning in PEH.

### 4.5 Illustrative application of the framework

The following example is provided only to illustrate how the framework may be applied in PEH teaching; it does not constitute empirical validation. Its purpose is therefore not to demonstrate effectiveness, but to show the analytical use of the framework in organizing the relationship among PEH content, embodied participation, health-related inquiry, teacher mediation, and GenAI-supported resources. One possible interdisciplinary theme is “basketball learning, tactical decision-making, and healthy participation.” This theme is appropriate because basketball lessons involve not only motor skill practice, such as passing, dribbling, shooting, and defensive movement, but also tactical understanding, spatial awareness, communication, cooperation, safety awareness, and sustained participation. It therefore reflects the integrative nature of PEH, where bodily practice can be connected with cognitive understanding, health-related reflection, and social learning.

In applying the framework, the teacher first aligns the theme with curriculum expectations related to movement competence, tactical awareness, health literacy, and collaborative participation. Learning goals may include performing basic basketball skills, understanding simple tactical principles such as spacing and passing options, recognizing safe and appropriate participation, and reflecting on how communication and cooperation influence game play. Content and tasks can then be organized around progressive movement activities, small-sided games, tactical scenarios, peer observation, and reflective discussion. During inquiry, students may compare different game situations, discuss why certain passing or movement choices are more effective, and reflect on how physical effort, teamwork, and decision-making shape participation in basketball. GenAI can support this process by generating age-appropriate tactical scenarios, simplified explanations of basketball rules, differentiated practice tasks, observation checklists, reflective prompts, and draft formative feedback. However, these outputs should be treated as provisional teaching resources. The teacher remains responsible for verifying the accuracy of AI-generated content, adapting tasks to students’ skill levels and physical conditions, observing movement quality and safety, and guiding students’ interaction during practice. In this way, the example illustrates how GenAI may support interdisciplinary thematic learning in PEH while remaining subordinate to pedagogical purpose, teacher judgment, embodied observation, and students’ active participation.

## 5 Discussion

### 5.1 Conceptual contribution

This review extends current discussions on GenAI by situating it within the specific pedagogical conditions of PEH. Previous AI-related research in PEH has often emphasized movement analysis, performance monitoring, skill evaluation, and technical optimization (6, 57, 58). These studies have advanced data-informed teaching and assessment, yet they offer limited guidance on how AI, and GenAI in particular, may support curriculum design, interdisciplinary learning, and broader educational aims in PEH. The present review addresses this gap by proposing a pedagogical framework that links GenAI use with curriculum alignment, content and task design, inquiry support, formative assessment, and human-AI collaboration.

The main conceptual contribution of this study lies in positioning GenAI as a pedagogically mediated resource in PEH. This framing moves the discussion beyond the use of GenAI for material generation or instructional efficiency and places greater emphasis on its integration into embodied, health-oriented, and socially situated learning processes. Within PEH, GenAI becomes educationally meaningful when it helps teachers connect movement practice, health knowledge, reflective inquiry, and social participation within coherent thematic learning experiences. Compared with more general frameworks of GenAI in education, the present framework is PEH-specific in three respects. First, it treats embodied participation as a central source of evidence for teaching judgment. PEH teachers need to interpret students’ movement quality, fatigue, confidence, coordination, peer interaction, and safety risks in real time, many of which cannot be fully captured through text-based AI outputs or pre-generated instructional suggestions. Second, the framework connects GenAI-supported inquiry with movement competence, health literacy, reflective decision-making, and socially situated participation. This expands the focus from content generation, cognitive scaffolding, or personalized learning toward the integrated development of embodied, cognitive, health-related, and social outcomes. Third, the framework positions teacher mediation as a safety- and value-sensitive process because PEH involves bodily activity, affective engagement, health-related choices, and interpersonal interaction. In this sense, the framework specifies how GenAI use can be pedagogically filtered through the embodied, relational, and health-promoting logic of PEH.

The framework also clarifies why interdisciplinary thematic learning is relevant to GenAI-supported PEH. In this context, interdisciplinary learning refers to the purposeful connection of bodily practice with health knowledge, scientific concepts, reflective decision-making, and social participation. This interpretation aligns with broader views of schools and quality physical education as important settings for developing life skills, positive behavior, and healthy patterns of living (59, 60). GenAI may support this process by helping teachers organize thematic content, generate guiding questions, provide differentiated resources, and structure formative feedback. These functions gain pedagogical value when they are aligned with PEH curriculum goals, students’ developmental needs, and teachers’ professional judgment. Therefore, the framework contributes to the literature by clarifying what GenAI may support in PEH and the pedagogical conditions under which such support may become educationally meaningful.

### 5.2 Implications for PEH curriculum and pedagogy

The proposed framework suggests that GenAI use in PEH should begin with curriculum purpose. Teachers need to identify the educational value of a theme, clarify the relationship between PEH goals and interdisciplinary learning goals, and decide what type of AI support is appropriate for the learning context. In this sense, GenAI can assist with planning, resource generation, scenario design, differentiated prompts, and formative feedback. These functions are useful only when teachers adapt them to students’ age, physical condition, learning needs, and classroom realities.

The framework also supports a more integrated approach to PEH content. Themes such as basketball learning and healthy participation, active lifestyles, injury prevention, physical activity and mental well-being, or digital health literacy can connect movement experience with health reasoning and social learning. Through such themes, students can move from performing activities to understanding why movement matters, how participation can be made safe and meaningful, and how physical activity relates to everyday choices.

Assessment also requires broader attention. Interdisciplinary thematic learning in PEH cannot be captured only through immediate performance outcomes. It also involves conceptual understanding, health-related reasoning, participation in inquiry, reflective awareness, collaboration, and social engagement. GenAI may help teachers organize learning evidence and draft formative feedback, but interpretation should remain grounded in teacher observation and curriculum goals. This point is consistent with work in GenAI and health-related education emphasizing pedagogical framing, faculty judgment, and meaningful learning design (61, 62).

### 5.3 Responsible and teacher-mediated implementation

Responsible implementation is especially important in PEH because learning takes place through bodily movement, peer interaction, emotional engagement, and real-time safety decisions. GenAI can generate explanations, tasks, and feedback, but teachers must judge whether these outputs are accurate, developmentally appropriate, and safe for students. This is particularly important when AI outputs involve movement correction, exercise suggestions, health explanations, or differentiated activity plans.

Teacher mediation also matters for preserving the relational and embodied qualities of PEH. Students’ fatigue, confidence, hesitation, coordination, and motivation are often visible through situated observation rather than written responses or data traces. These dimensions require professional judgment and responsive interaction. GenAI may extend teachers’ planning and feedback capacity, but it cannot interpret the full complexity of students’ embodied participation.

Ethical and equity issues add another layer of responsibility. Reliability, bias, privacy, digital access, and data security affect whether AI-supported PEH is trustworthy and inclusive. Unequal access to devices, connectivity, and digital literacy may reproduce existing educational and health inequalities. Concerns about AI use in K-12 and health-related education also suggest that institutions need clearer guidance on responsibility, data use, and appropriate pedagogical boundaries (63–66). For PEH, such guidance should include the verification of health-related content, safe use of AI-generated activity suggestions, and protection of student data.

### 5.4 Limitations and future research

This study has several limitations. First, it is a narrative review and conceptual framework, and the proposed model has not yet been empirically tested. Its contribution therefore lies in conceptual clarification and pedagogical orientation rather than in demonstrated instructional effectiveness. Future work should examine whether the framework can support teaching and learning in authentic PEH settings. Second, although this review used structured search, eligibility, and synthesis procedures, it was not designed as a systematic or scoping review. A formal critical appraisal of the included studies was not conducted, and the literature selection may be subject to bias. The rapid development of GenAI also means that new studies may emerge quickly after the review period. Third, the current evidence base remains uneven. Many GenAI-related PEH studies are conceptual, practice-oriented, short-term, or concentrated in higher education contexts. Evidence from primary and secondary PEH classrooms, diverse cultural settings, and long-term instructional practice remains limited. The illustrative application included in this paper should also not be interpreted as evidence that the framework has been validated. It only shows how the framework may be applied pedagogically.

Future research should examine how GenAI-supported interdisciplinary thematic learning operates in authentic PEH settings. Design-based studies, classroom interventions, and longitudinal research are needed to explore how teachers interpret and adapt AI-generated resources, how students experience human-AI collaboration during embodied learning, and how such approaches influence movement competence, health literacy, reflective decision-making, collaboration, and motivation. Further work should also examine assessment design, teacher readiness, ethical guidance, and institutional conditions for sustainable implementation. These directions are essential for understanding whether GenAI can support PEH in ways that are pedagogically meaningful, ethically responsible, and aligned with health-promoting educational aims.

## 6 Conclusion

GenAI has created new possibilities for teaching and learning, but its value in PEH depends on pedagogical integration and meaningful classroom use. By synthesizing PEH-related and interdisciplinary learning literature, this study identified a shift from performance-oriented AI applications toward more pedagogically grounded uses of GenAI, while also highlighting the need for clearer guidance on interdisciplinary thematic learning in PEH. The proposed framework presents GenAI as a teacher-mediated resource that may help connect bodily practice, health knowledge, reflective inquiry, and social participation. Within this framework, teachers play a central role in curriculum alignment, embodied observation, contextual interpretation, safety judgment, and meaningful learning design. When implemented carefully and contextually, GenAI may support classroom innovation, health literacy, health education, and health-promoting PEH practice. The central implication of this review is that the value of GenAI in PEH should be judged by whether it helps teachers connect movement, health understanding, reflection, and social participation in safe, meaningful, and pedagogically coherent ways. Future empirical research is needed to test and refine the framework in authentic PEH settings.
